# Optimising Clinical Trial Design in Older Cancer Patients

**DOI:** 10.3390/geriatrics3030034

**Published:** 2018-06-22

**Authors:** Shóna Whelehan, Orlaith Lynch, Niall Treacy, Ciara Gleeson, Andrea Oates, Anita O’Donovan

**Affiliations:** Discipline of Radiation Therapy, School of Medicine, Trinity College Dublin, Dublin, Ireland; whelehs@tcd.ie (S.W.); orlynch@tcd.ie (O.L.); treacyni@tcd.ie (N.T.); gleesoc3@tcd.ie (C.G.); oatesan@tcd.ie (A.O.)

**Keywords:** oncology, clinical trials, geriatric oncology, design, endpoints, quality of life

## Abstract

Cancer is predominantly a disease of older patients, with over half of those aged over 65 years of age being diagnosed with cancer at some stage. Despite comprising a significant proportion of the patients that we see in clinical practice, there is a lack of representation of older patients in cancer clinical trials. This is mainly due to restrictive trial inclusion criteria that prevent older patients from participating. Also, trial endpoints, such as overall survival, may not represent the most important and most meaningful endpoints for older patients. The latter may place more significance on quality of life and other outcomes such as functional independence. Baseline assessment using Comprehensive Geriatric Assessment, may provide a better framework for quantifying patient outcomes for varying degrees of fitness or frailty. This short communication makes the case for more age appropriate endpoints, such as quality of life, toxicity and functional independence, and that novel trial designs are necessary to inform evidence-based care of older cancer patients.

## 1. Introduction

Cancer is a disease of the ageing population, with over half of those diagnosed each year, in countries such as Ireland, aged 65 years of age and older [1]. Despite constituting a large proportion of cancer patients, quality research is lacking to support the optimum treatment approach for the older patient population. This is due to the under-representation and poor enrolment of older patients in clinical trials [2,3,4]. Reasons for this may include: functional reserve decline, an escalation of comorbid conditions, absence of social support systems and decreased access to clinical trials. Fang et al. evaluated how treatment of older cancer patients often deviates from guideline concordant care, with some receiving “de-intensified” regimens, especially those receiving curative treatment of advanced disease [5]. However, numerous trials have shown that older patients derive similar benefit from oncology drug trials as their younger counterparts [6,7,8]. Enhanced clinical trial design has the potential to establish optimal standards in geriatric oncology, tailoring treatments to patients based on their co-morbidities and functional reserve capacity.

There is no widely accepted definition of an “older” or “elderly” person. The United Nations generally uses 60+ years as a cutoff to define old age; however most developed countries now accept 65+ years as a more suitable definition of old [9,10]. This 65-year threshold, or even 70 [11], is used in many scientific publications and clinical trials. However, it should be noted that, although approximately 60% of new cancer cases occur in the elderly, they comprise only a quarter of participants in cancer clinical trials [12]. The use of a chronological age to mark the advent of “older” age assumes equivalence with physiological age, yet these two are clearly not synonymous.

Previous studies have several pitfalls and limitations in trial design, regarding their ability to inform the care of older patients. Consequently, older patients are often misrepresented and results are thus misinterpreted [13]. Age-related bias can attribute to the delay in significant evidence based practice in geriatric oncology. There have been some discrepancies in the identification of “older” populations [14]. Trials have been designed to assume that physiological age will play no role in the response of the patient to treatment. To avoid ageist assumptions in clinical trials, the physiological age of a patient should be a factor that influences their treatment decision. Decline in physiological reserves may be characterized via common frailty measures, such as phenotypic criteria [15] or cumulative deficit models [16,17].

Conversely, a heterogeneous patient population in clinical trials might be considered a truer representation of reality. Clinically, patients will present with variable co-morbidities, performance status, frailty and physiological age. Evidently, characterising the heterogeneity of the older cancer patient cohort, and their ability to tolerate oncologic treatment, is extremely difficult without the aid of clinical trial information to guide clinicians. Targeting designs that consider a larger population size, baseline and ongoing Comprehensive Geriatric Assessment (CGA), defined end-goals, age-specific outcomes, of which Quality of Life (QoL) and functional status is key. Through critical analysis of future research, further deductions can be made on the varying responses to treatment by a heterogeneous aging population.

## 2. Implementation of Comprehensive Geriatric Assessment

CGA is used clinically to assess an older patient’s cognitive function, nutritional status, co-morbidities, physical function, psychological function and incorporates the patient’s social support system. It can determine a patient’s overall health status by detecting and possibly addressing cumulative deficits. CGA answers the question, is the patient fit, vulnerable or frail [17]? This can potentially inform trial design, both as selection criteria and outcome measure. The inclusion of CGA for older cancer patients has been recommended by multiple organisations that highlight critical endpoints and aspects of trial design in older patients [18,19]. The absence of CGA in a clinical trial can make it difficult to determine whether the inclusion criteria consisted of fit patients only, or a combination of fit as well as frail patients. Eastern Cooperative Oncology Group (ECOG) based inclusion criteria have been shown to poorly represent the full depiction of frailty in the older patient population [20]. Therefore, the data collected may not relate well to the general older population in clinical practice.

A CGA can also help to stratify older patients into different treatment arms of clinical trials. CGA results could be interpreted to allocate patients to various treatment regimes for clinical trials, as opposed to chronological age-based allocation. The Elderly Selection on Geriatric Index Assessment (ESOGIA) phase III randomised control trial compared age-based treatment allocation versus CGA-based allocation in older patients with advanced lung cancer [21]. Although CGA-based allocation did not significantly improve overall survival, it reduced treatment toxicity which is an important endpoint in trial design for older patients. The phase III ELderly heAd and Neck cancer-Oncology eValuation (ELAN-ONCOVAL) trial used a Suited Geriatric Evaluation (SGE), derived from the CGA, to allocate patients into treatment arms depending on whether they were fit or unfit [22]. This study concluded that a CGA-based SGE tool was necessary for optimal care and resulted in the addition or elimination of treatment options for many patients based on their frailty status. Perhaps the inclusion of a standard cancer specific geriatric assessment tool in the future could be beneficial for cross comparison between trials. Unfortunately, there is a lack of standardized assessments worldwide which makes comparison between studies difficult at present [23,24]. A specific assessment tool could also be useful in tailoring treatment, as seen in older patients with head and neck cancer [25].

## 3. Clinical Trial Methodology

There is a great need for novel trial designs in geriatric oncology research. Although survival (overall/progression-free) is recognised as the most valuable outcome of any clinical trial, questions have been raised regarding its significance in older patients, particularly due to patient comorbidities which are likely to be a contributing factor to patient death. A workshop held by the European Organisation for Research and Treatment of Cancer (EORTC) in 2011 [26] explored methods of improving clinical design and suggested looking at alternative endpoints, such as QoL, toxicity and functional independence. Measurement of disease-specific survival could also be useful when evaluating treatments for this patient group as it would indicate the number of patients who actually died as a result of their cancer versus other chronic conditions.

Clinical trial inclusion criteria is an area of much debate. Chronological age, CGA and medical criteria may all be used as the basis for inclusion criteria [26]. A considerable amount of clinical trials in oncology use an age of greater than 70 years as an appropriate definition of the ‘older’ patient, as per recent International Society of Geriatric Oncology (SIOG) recommendations [11]. A position statement from the U13 conference in 2012 [18] suggests methods of improving inclusion criteria in clinical trials for older patients. They believe irrelevant exclusion criteria should be omitted as fewer inclusion and exclusion criteria are preferable in order to eliminate patient selection bias. Traditional exclusion criteria based on age, performance status and stringent organ function restrictions have been unhelpful in adding to the evidence base for the management of older cancer patients [27].

With such limited literature available in the area of geriatric oncology, more focused trials are called for in order to provide more reliable conclusions about suitable treatments for specific treatment sites. Also, notable differences in treatment patterns between older and younger patients are evident in the literature [28,29]. Clinicians may fear induction of significant toxicity if giving standard doses to older patients. The geriatric oncology literature offers a potential compromise and suggests a ‘start low and go slow’ approach, which involves initial administration of a reduced dose and if the patient tolerates this well, increasing the dose to standard level [30]. This was successfully used in the Medical Research Council Fluorouracil, Oxaliplatin, CPT11 [irinotecan]: Use and Sequencing 2 (MRC-FOCUS2) trial involving older adults with metastatic colorectal cancer [31].

## 4. Quality of Life Endpoints

A patient-centred approach is crucial. While therapy may increase survival rates, a reduction in patient’s health-related QoL potentially negates any benefits. Sekeres et al. state that QoL was more important to patients than the duration of life when making treatment decisions [32]. Another study showed that maintenance of physical and cognitive function was more important to older patients than traditional survival endpoints [33]. Survivors of haematological cancer are shown to be at increased risk of depression and a variety of physical impairments compared to a control population [34]. Tools for measuring QoL such as the elderly specific EORTC QLQ-ELD15 [35] and Q–TWIST (Quality-Adjusted Time Without Symptoms or Toxicity) [36] can be used to determine time with/without significant toxicity experienced until death and can be used to compare treatments.

A patient’s QoL should be prioritised as a treatment outcome in the older patient group. Although overall survival is an important outcome in most patient groups, compromising a patient’s QoL to achieve this could cause the patient to lose his or her independence at home or lose the ability to carry out daily activities. Although CGA-based allocation does not significantly improve overall survival, it has been proven to reduce toxicity and thus results in a better QoL for the patient after treatment, which is a valuable endpoint in clinical trial design for older patients [2,3]. CGA should be used to assess the patient’s frailty status and estimate the severity of expected side effects and the impact these would have on a patient’s QoL. Should the patient be rendered too frail, their QoL could be negatively impacted or their functional reserve could be depleted after intense treatment. Overall survival should not be the only goal when designing clinical trials for older patients as QoL is equally as important, and with regular CGA assessment throughout the course of treatment the patient’s frailty status can be monitored.

## 5. Conclusions

Demographic changes in most countries mandate an urgent change in clinical trial design in order to better support the treatment of older patients with cancer. Under-representation of older patients is a significant issue that needs to be addressed. A recent survey of the 11,000 strong membership of the Alliance for Clinical Trials in Oncology members in the US provided the following recommendations for improvement of clinical trial enrollment for older adults [37]. 1. Create more trials dedicated solely to older patients, 2. minimise exclusion criteria based on comorbidities in clinical trials, 3. consider strategies to increase enrollment for those aged 65/70 and older and 4. require that most/all Alliance trials include an ‘expansion cohort’ of older patients, and analysis of outcomes/toxicity/QoL for this older patient group. CGA should be incorporated into trial design in order to stratify treatments based on varying degrees of fitness or frailty. It is important to have research based guidelines on the effects of treatment on cancer outcome and toxicity, QoL impairment and frailty. Therefore, more age appropriate endpoints, such as QoL, toxicity and functional independence and more novel trial designs are necessary in order to inform evidence-based care of older cancer patients.

## References

[1] (2017). Cancer Factsheet: Overview & Most Common Cancers [Internet].

[2] Scher K.S., Hurria A. (2012). Under-representation of older adults in cancer registration trials: Known problem, little progress. J. Clin. Oncol. Off. J. Am. Soc. Clin. Oncol..

[3] Talarico L., Chen G., Pazdur R. (2004). Enrollment of elderly patients in clinical trials for cancer drug registration: A 7-year experience by the us food and drug administration. J. Clin. Oncol..

[4] Younger E., Litiere S., Le Cesne A., Mir O., Gelderblom H., Italiano A., Marreaud S., Jones R.L., Gronchi A., van der Graaf W.T.A. (2018). Outcomes of elderly patients with advanced soft tissue sarcoma treated with first-line chemotherapy: A pooled analysis of 12 eortc soft tissue and bone sarcoma group trials. Oncologist.

[5] Fang P., He W., Gomez D.R., Hoffman K.E., Smith B.D., Giordano S.H., Jagsi R., Smith G.L. (2017). Influence of age on guideline-concordant cancer care for elderly patients in the united states. Int. J. Radiat. Oncol. Biol. Phys..

[6] Arciero V.S., Cheng S., Mason R., McDonald E., Saluja R., Chan K.K.W. (2018). Do older and younger patients derive similar survival benefits from novel oncology drugs? A systematic review and meta-analysis. Age Ageing.

[7] Zhao C.J., Li S., Liu Q. (2018). Meta-analysis of molecular targeted agents in the treatment of elderly patients with metastatic colorectal cancer: Does the age matter?. J. Cancer Res. Ther..

[8] Buechel M., McGinnis A., Vesely S.K., Wade K.S., Moore K.N., Gunderson C.C. (2018). Consideration of older patients for enrollment in phase 1 clinical trials: Exploring treatment related toxicities and outcomes. Gynecol. Oncol..

[9] World Health Organisation (WHO) Health Statistics and Health Information Systems. Definition of an Older or Elderly Person. https://www.scribd.com/document/190077600/WHO-Definition-of-an-Older-or-Elderly-Person.

[10] Gorman M., Judith R., Ewing T. (2017). Development and the rights of older people. The Ageing and Development Report: Poverty, Independence and the World‘s Older People.

[11] Wildiers H., Heeren P., Puts M., Topinkova E., Janssen-Heijnen M.L., Extermann M., Falandry C., Artz A., Brain E., Colloca G. (2014). International society of geriatric oncology consensus on geriatric assessment in older patients with cancer. J. Clin. Oncol..

[12] Lewis J.H., Kilgore M.L., Goldman D.P., Trimble E.L., Kaplan R., Montello M.J., Housman M.G., Escarce J.J. (2003). Participation of patients 65 years of age or older in cancer clinical trials. J. Clin. Oncol..

[13] Lichtman S.M. (2012). Clinical trial design in older adults with cancer—The need for new paradigms. J. Geriatr. Oncol..

[14] Hurria A., Lachs M.S., Cohen H.J., Muss H.B., Kornblith A.B. (2006). Geriatric assessment for oncologists: Rationale and future directions. Crit. Rev. Oncol. Hematol..

[15] Fried L.P., Tangen C.M., Walston J., Newman A.B., Hirsch C., Gottdiener J., Seeman T., Tracy R., Kop W.J., Burke G. (2001). Frailty in older adults: Evidence for a phenotype. J. Gerontol. Ser. A Biol. Sci. Med. Sci..

[16] Clegg A., Young J., Iliffe S., Rikkert M.O., Rockwood K. (2013). Frailty in elderly people. Lancet.

[17] Balducci L., Extermann M. (2000). Management of cancer in the older person: A practical approach. Oncologist.

[18] Wildiers H., Mauer M., Pallis A., Hurria A., Mohile S.G., Luciani A., Curigliano G., Extermann M., Lichtman S.M., Ballman K. (2013). End points and trial design in geriatric oncology research: A joint european organisation for research and treatment of cancer–alliance for clinical trials in oncology–international society of geriatric oncology position article. J. Clin. Oncol..

[19] Soto-Perez-De-Celis E., Lichtman S.M. (2017). Considerations for clinical trial design in older adults with cancer. Expert Opin. Investig. Drugs.

[20] Kelly C.M., Shahrokni A. (2016). Moving beyond karnofsky and ECOG performance status assessments with new technologies. J. Oncol..

[21] Corre R., Greillier L., Le Caer H., Audigier-Valette C., Baize N., Berard H., Falchero L., Monnet I., Dansin E., Vergnenegre A. (2016). Use of a comprehensive geriatric assessment for the management of elderly patients with advanced non-small-cell lung cancer: The phase iii randomized esogia-gfpc-gecp 08-02 study. J. Clin. Oncol. Off. J. Am. Soc. Clin. Oncol..

[22] Mertens C., Le Caer H., Ortholan C., Blot E., Even C., Rousselot H., Peyrade F., Sire C., Cupissol D., Pointreau Y. (2017). 1050pdthe elan-oncoval (elderly head and neck cancer-oncology evaluation) study: Evaluation of the feasibility of a suited geriatric assessment for use by oncologists to classify patients as fit or unfit. Ann. Oncol..

[23] O’Donovan A., Mohile S.G., Leech M. (2015). Expert consensus panel guidelines on geriatric assessment in oncology. Eur. J. Cancer Care.

[24] Mohile S.G., Velarde C., Hurria A., Magnuson A., Lowenstein L., Pandya C., O’Donovan A., Gorawara-Bhat R., Dale W. (2015). Geriatric assessment-guided care processes for older adults: A delphi consensus of geriatric oncology experts. J. Natl. Compr. Cancer Netw. JNCCN.

[25] Pottel L., Lycke M., Boterberg T., Pottel H., Goethals L., Duprez F., Van Den Noortgate N., De Neve W., Rottey S., Geldhof K. (2014). Serial comprehensive geriatric assessment in elderly head and neck cancer patients undergoing curative radiotherapy identifies evolution of multidimensional health problems and is indicative of quality of life. Eur. J. Cancer Care.

[26] Pallis A.G., Ring A., Fortpied C., Penninckx B., Van Nes M.C., Wedding U., vonMinckwitz G., Johnson C.D., Wyld L., Timmer-Bonte A. (2011). Eortc workshop on clinical trial methodology in older individuals with a diagnosis of solid tumors. Ann. Oncol..

[27] Hamaker M.E., Stauder R., van Munster B.C. (2014). Exclusion of older patients from ongoing clinical trials for hematological malignancies: An evaluation of the national institutes of health clinical trial registry. Oncologist.

[28] Hurria A., Wong F.L., Villaluna D., Bhatia S., Chung C.T., Mortimer J., Hurvitz S., Naeim A. (2008). Role of age and health in treatment recommendations for older adults with breast cancer: The perspective of oncologists and primary care providers. J. Clin. Oncol..

[29] Berry M.F., Worni M., Pietrobon R., D’amico T.A., Akushevich I. (2013). Variability in the treatment of elderly patients with stage iiia (n2) non–small-cell lung cancer. J. Thorac. Oncol..

[30] Hurria A., Dale W., Mooney M., Rowland J.H., Ballman K.V., Cohen H.J., Muss H.B., Schilsky R.L., Ferrell B., Extermann M. (2014). Designing therapeutic clinical trials for older and frail adults with cancer: U13 conference recommendations. J. Clin. Oncol..

[31] Seymour M.T., Thompson L.C., Wasan H.S., Middleton G., Brewster A.E., Shepherd S.F., O’Mahony M.S., Maughan T.S., Parmar M., Langley R.E. (2011). Chemotherapy options in elderly and frail patients with metastatic colorectal cancer (mrc focus2): An open-label, randomised factorial trial. Lancet.

[32] Sekeres M., Stone R., Zahrieh D., Neuberg D., Morrison V., De Angelo D., Galinsky I., Lee S. (2004). Decision-making and quality of life in older adults with acute myeloid leukemia or advanced myelodysplastic syndrome. Leukemia.

[33] Fried T.R., Bradley E.H., Towle V.R., Allore H. (2002). Understanding the treatment preferences of seriously ill patients. N. Engl. J. Med..

[34] Gotze H., Kohler N., Taubenheim S., Lordick F., Mehnert A. (2018). Polypharmacy, limited activity, fatigue and insomnia are the most frequent symptoms and impairments in older hematological cancer survivors (70+): Findings from a register-based study on physical and mental health. J. Geriatr. Oncol..

[35] Johnson C., Fitzsimmons D., Gilbert J., Arrarras J.-I., Hammerlid E., Bredart A., Ozmen M., Dilektasli E., Coolbrandt A., Kenis C. (2010). Development of the european organisation for research and treatment of cancer quality of life questionnaire module for older people with cancer: The eortc qlq-eld15. Eur. J. Cancer.

[36] Schwartz C., Cole B., Vickrey B., Gelber R. (1995). The q-twist approach to assessing health-related quality of life in epilepsy. Qual. Life Res..

[37] Freedman R.A., Dockter T.J., Lafky J.M., Hurria A., Muss H.J., Cohen H.J., Jatoi A., Kemeny M.M., Ruddy K.J. (2018). Promoting accrual of older patients with cancer to clinical trials: An alliance for clinical trials in oncology member survey (a171602). Oncologist.

